# Reply to Asim Armagan Aydin, Erkan Kayikcioglu, and Ramazan Oguz Yuceer’s Letter to the Editor re: Muhammet Demirbilek, Göktuğ Kalender, Said Bıyıkoglu, et al. External Validation of Nomograms for Predicting Pelvic Lymph Node Metastases in Patients with Prostate Cancer and the Added Value of the Prostate-specific Membrane Antigen Positron Emission Tomography–based PRIMARY Score. Eur Urol Open Sci 2025;82:170–7

**DOI:** 10.1016/j.euros.2026.01.002

**Published:** 2026-01-22

**Authors:** Bülent Önal, Muhammet Demirbilek, Levent Kabasakal

**Affiliations:** a Department of Urology, Cerrahpasa Faculty of Medicine, Istanbul University-Cerrahpasa, Istanbul, Turkey; b Department of Urology, Dr. Yusuf Azizoglu State Hospital, Diyarbakir, Turkey; c Department of Nuclear Medicine, Cerrahpasa Faculty of Medicine, Istanbul University-Cerrahpasa, Istanbul, Turkey

We thank our esteemed colleagues for their encouraging comments on our study [1]. The 2023 European Association of Urology guidelines recommended nomograms to guide extended pelvic lymph node dissection (ePLND) [2], but the 2025 guidelines no longer include these nomograms and advise performing ePLND whenever lymph node dissection is indicated [3]. Although the role of PLND remains a matter of debate, recent studies have shown that ePLND may offer clinical benefits: Touijer et al. [4] reported that ePLND has a protective effect against metastasis, and Furrer et al. [5] found that ePLND was associated with better metastasis-free survival. These findings suggest that ePLND and pelvic lymph node metastases (pN1 stage) continue to hold prognostic relevance.

Despite their widespread use in lymph node staging, imaging techniques may still offer limited accuracy. Therefore, predictive models such as nomograms remain valuable tools for reliably assessing pN1 status. Although prostate-specific membrane antigen (PSMA)-based positron emission tomography (PET) is highly specific and widely adopted for nodal staging, it may be insufficient when used in isolation. In this context, radiomics features and semiquantitative parameters (eg, PRIMARY score, maximum and mean standardized uptake values, intraprostatic PSMA tumor volume) derived from PSMA PET, as well as the biological properties of PSMA, are considered promising tools for improving the detection of pN1 disease. Recent studies have shown that nomograms that include PSMA PET parameters may outperform traditional nomograms in predicting nodal metastases [6], [7]. At this stage, both the spatial patterns of intraprostatic PSMA ligand uptake and their integration into predictive nomograms may serve as valuable tools to enhance staging accuracy. In addition, Akcay et al. [8] reported that the PSMA PET–based PRIMARY score may serve as an indicator of tumor aggressiveness and assist in decision-making for active surveillance. As with pN1 stage, this finding reflects the influence of tumor biology on PSMA uptake and supports its potential role as a marker of tumor aggressiveness, which is an unsurprising but important conclusion.

As we noted in our study, positive PSMA PET findings may prompt surgeons to perform more extensive PLND in selected cases, and thus increase the likelihood of detecting lymph node metastasis [1]. Although this may appear to introduce bias, it actually highlights the clinical utility of PSMA PET imaging. Moreover, although addition of the PRIMARY score led to only marginal improvements in the area under the receiver operating characteristic curve, results for the likelihood ratio test showed that the new model provided a statistically significant improvement (*p* < 0.001) [1]. This model should be evaluated for statistical validity and tested for clinical benefit in larger, prospective cohorts. Karpinski et al. [9] analyzed PROMISE registry data for 1889 patients who underwent PSMA PET imaging for staging between 2012 and 2021. In their cohort, which included 231 deaths, a PRIMARY score of 5 was associated with shorter overall survival. This finding underscores the potential association between PRIMARY scores and oncological outcomes in prostate cancer.

Despite its increasing clinical adoption, access to PSMA PET across Europe remains heterogeneous, with substantial variation in reimbursement policies, cost structures, and billing procedures between and within countries [10]. These disparities highlight the practical limitations that persist in real-world implementation of PSMA/PET.

In conclusion, patterns of intraprostatic uptake of PSMA ligands on PET, lymph node staging, and nomograms that include these features may contribute to more accurate nodal staging. Prospective multicenter studies are essential to demonstrate the actual clinical benefit of these approaches.

  ***Conflicts of interest***: The authors have nothing to disclose.
